# Longitudinal associations between preschoolers’ 24-h movement behaviors and development of self-regulation, executive function, vocabulary, and numeracy

**DOI:** 10.1186/s12889-025-24605-z

**Published:** 2025-10-06

**Authors:** Kristoffer Buene Vabø, Katrine Nyvoll Aadland, Anthony D. Okely, Caterina Pesce, Eivind Aadland

**Affiliations:** 1 Department of Sport, Food and Natural Sciences, Faculty of Education, Arts and Sports, Western Norway University of Applied Sciences, Sogndal, Norway; 2 Early Start and School of Education, University of Wollongong, Wollongong, NSW Australia; 3 Department of Movement, Human and Health Sciences, University of Rome “Foro Italico”, Rome, Italy

**Keywords:** Early childhood, Physical activity, Sleep, Screen time, Cognitive function, Academic skills

## Abstract

**Background:**

Physical activity (PA), sedentary behavior (SED), including screen time, and sleep constitute 24-h “movement behaviors” and may influence development of children’s cognitive function and academic skills. However, the existing longitudinal evidence in young children is scarce and inconsistent. The aim of this study was to examine longitudinal associations between these movement behaviors and change in self-regulation, executive function, vocabulary, and numeracy in preschool children.

**Methods:**

561 children (53% boys; aged 3–4 years at baseline) from the Active Learning Norwegian Preschool(er)s study provided data on PA and SED (ActiGraph GT3X +), screen time (parent-report), and sleep (parent-report) at baseline, and at least one outcome variable: self-regulation (Head-Toes-Knees-Shoulders task), inhibition, working memory, cognitive flexibility, vocabulary, or numeracy (Early Years Toolbox) at baseline and 18-month follow-up. We analyzed longitudinal associations between movement behaviors at baseline and change in cognitive and academic outcomes between baseline and follow-up using multivariate pattern analysis and linear regression analysis.

**Results:**

We found no significant associations in the primary analyses. Subgroup analyses showed significant associations in girls for change in inhibition (R^2^ = 5.40%) and self-regulation (R^2^ = 4.22%). For inhibition, associations were negative for SED (0–99 cpm) and positive for all PA intensities (≥ 100 counts per minute). For self-regulation, associations were positive for SED and negative for all PA intensities. Using linear regression, we found no significant associations for separate explanatory variables or for compliance with all movement behavior guidelines with cognitive or academic outcomes.

**Conclusion:**

Generally, young children’s movement behaviors were not associated with change in self-regulation, executive function, vocabulary, or numeracy during preschool years.

**Trial registration:**

The study was approved by the institutional ethics committee and the Norwegian Centre for Research Data (reference number: 248220), and registered at clinicaltrials.gov August 7, 2019, with the identifier NCT04048967.

**Supplementary Information:**

The online version contains supplementary material available at 10.1186/s12889-025-24605-z.

## Background

Physical activity (PA), especially moderate-to-vigorous-intensity PA (MVPA), plays a key role in preventing noncommunicable diseases and is important for children’s optimal health and development [1, 2]. Young children’s MVPA only accounts for a small portion (< 5%) of the 24-h day, with sedentary behavior (SED, ~ 40%), included screen time, sleep (~ 40%), and light-intensity PA (LPA, ~ 15%) constituting the major time components of the day [3]. These behaviors have collectively been referred to as “movement behaviors” and are comprised in the World Health Organization (WHO) guidelines for children from birth to age 5 [4]. It is understood that high levels of PA and sleep, and low levels of SED and screen time are the most ideal combination of movement behaviors for optimal health in young children [5].

Cognitive functions, especially the processes of self-regulation and executive function (EF) [6], are important health and development indicators at an early age [7–9]. Self-regulation is generally defined as the ability to control thoughts, overt behaviors, and emotions to achieve goal-directed behaviors [10]. Self-regulation conceptually includes EF processes, a family of top-down mental processes needed to stay focused, store and process information, and flexibly adapt to varying challenges [11, 12]. Therefore, self-regulation and EF are important for young children’s learning and academic skills, and their development is considered an early life marker for later life successes [13–16]. PA involve physical effort and cognitive engagement that are suggested to cause physiological changes to brain regions critical to cognitive development and learning [17, 18]. Also, sleep may play a facilitative or direct physiological role for cognition, while screen time may act as a direct displacement of sleep, PA, and learning behaviors [18–20]. However, there is limited evidence regarding both the separate and combined influences of movement behaviors for change in different cognitive and academic outcomes in young children [1, 7, 9, 21–23].

Most previous studies investigating associations between movement behaviors and cognitive function and academic skills in preschool-aged children have been cross-sectional and the findings are inconsistent [5, 9, 24–27]. Longitudinal evidence, needed to determine potential causal relationships, is scarce and generally show mixed or null associations for PA [9, 28, 29], negative associations for screen time [22, 30], and positive associations for sleep [20, 23] with cognitive and/or academic outcomes. However, more high-quality evidence is warranted [9, 23, 30]. Small studies, the use of dichotomized variables, reliance on parent-reported PA, and the selective inclusion of only a part of the PA intensity spectrum [28, 29, 31–33], may partly explain the inconsistent findings for PA. Several methods exists to determine the amount of time spent in various PA intensities, however, there exists no consensus on the optimal data collection and processing decisions for accelerometer data in preschool-aged children, including the choice of intensity cut-points [34, 35]. Thus, application of a traditional intensity cut-point approach summing time spent across the entire intensity spectrum in a few gross intensity categories may misclassify time use and, additionally, cause a loss of information [36]. Using the entire PA spectrum allows for analyzing higher resolution accelerometry data and obtaining a detailed PA intensity association pattern [37]. Furthermore, using triaxial accelerometry has been shown to be a more powerful approach than uniaxial accelerometry to reveal associations for PA with cardiometabolic health, body mass index (BMI), and physical fitness in children [38–40].

The primary aim of this study was to determine longitudinal multivariate association patterns for 24-h movement behaviors (the PA intensity spectrum, screen time, and sleep) at baseline with change in self-regulation, EF, vocabulary, and numeracy over 18 months in preschoolers. The secondary aims were to examine moderation by sex and to determine the combined influence of meeting or not meeting all movement behavior guidelines.

## Methods

### Design and participants

The present study used a longitudinal design, examining associations between movement behaviors (explanatory variables) and change in cognitive function and academic skills (outcome variables) over 18 months in preschoolers. Data were obtained from the Active Learning Norwegian Preschool(er)s (ACTNOW) cluster randomized controlled trial [41], conducted in Western Norway between August 2019 and June 2022 (baseline data collected during autumn 2019 and 2020 and follow-up data collected during spring 2021 and 2022). The main aim of the ACTNOW study was to investigate effects of a PA professional development intervention for preschool staff on children’s PA and developmental outcomes such as physical fitness, motor competence, socioemotional health, self-regulation, EF, and academic skills [41]. Fifty-six preschools were invited to participate, of which 46 preschools (82%), encompassing 1533 children, agreed to participate. At baseline, 1263 3–5-year-olds (born in 2015–2017, 82%) participated, of whom 820 children were aged 3–4 years (65%) and provided data for relevant outcomes at baseline and at the 18-month follow-up (i.e., 5-year-olds started school).

### Explanatory variables

#### Physical activity and sedentary behavior

We measured PA and SED using ActiGraph GT3X + accelerometers (ActiGraph, LLC, Pensacola, Florida, USA) [42]. Children were instructed to wear the accelerometer on their right hip 24 h a day for seven consecutive days, including while sleeping, except during water-based activities. Consecutive periods of ≥ 20 min of zero counts were defined as non-wear time [43]. A wear time of ≥ 480 min/day between 06:00 and 22:00 was the criterion for a valid day. Children providing ≥ 3 weekdays and ≥ 1 weekend day of valid data were included in the analyses [44]. The accelerometers were initialized at a sampling rate of 30 Hz and analyzed at 1-s epochs [45, 46] using a custom script in MATLAB (MathWorks, Massachusetts, USA).

For the primary analyses, we created 17 variables of time spent in intensity intervals (0–99, 100–999, 1000–1999, 2000–2999, … 14,000–14999, and ≥ 15,000 counts per minute [cpm]) [40] from each of the three axes, to capture movement in narrow intensity intervals across the triaxial intensity spectra. For the secondary analyses, descriptive purposes, and to guide our interpretation of associations, PA levels were reported as average total PA (TPA, cpm), and minutes per day spent in SED (≤ 100 cpm), LPA (101–2295 cpm), moderate-intensity PA (MPA, 2296–4011 cpm), vigorous-intensity PA (VPA, ≥ 4012 cpm), and MVPA (≥ 2296 cpm), as proposed by Evenson et al. [47] (vertical axis only). Children met the PA guidelines if they had an average of ≥ 180 min/day of total PA and ≥ 60 min/day of MVPA [4].

#### Screen time

We assessed children’s screen time at baseline by a parent(s)/guardian(s) questionnaire, adapted from the Sunrise study protocol [48]. Parents were asked to report the children’s total time (hours/min) spent on any electronic device (e.g., smartphone, tablet, video game, watching television/movies, or online), while sitting or lying down, on a typical 24-h day in the past week. Children met the screen time guidelines if they had ≤ 1 h/day of screen time [4].

#### Sleep

We assessed children’s sleep at baseline using a parent(s)/guardian(s) questionnaire adapted from the Sunrise study [48], where parents were asked to report their child’s sleep duration in hours and minutes on a typical 24-h day, including naps. In addition, since the validity of sleep questionnaires may be limited by parents’ recall bias, we also assessed children’s sleep using accelerometry that may objectively capture both nighttime sleep patterns and naps over several days [49–51]. For accelerometry-derived sleep, we analyzed children’s sleep in two steps using R Statistical Software (v4.3.1) [52]. In step 1, we used the *PhysicalActivity* (v0.2.4) package [53] to mark wear/non-wear periods, defined as ≥ 90 min of consecutive zero counts with up to 2 min of non-zero interruption. In step 2, we used the *PhysActBedRest* (v1.1) package [54], which contains an algorithm developed and validated to identify sleep/bedrest from waist-worn triaxial accelerometers in preschool children [55]. This algorithm uses a decision-tree approach to determine sleep/bedrest and has good sensitivity (0.94), specificity (0.97), and accuracy (0.95) when compared to visual identification of accelerometry-derived sleep periods. As recommended by Tracy et al. [55], we used vector magnitude and 60-s epochs to identify sleep periods. We included children with valid accelerometry data (480 min of wear time) between 22:00–05:59 for ≥ 3 weekdays and ≥ 1 weekend day. The average daily time spent sleeping between 18:00 one day and 17:59 the next day (to include daytime naps) was summed and averaged across valid days to determine sleep duration. Children met the sleep guidelines if they had a minimum sleep duration of 10 h/day [4].

### Outcome variables

Self-regulation was assessed by a structured, direct measure, the Head-Toes-Knees-Shoulders (HTKS) task [56]. The HTKS has shown construct validity in measuring children’s behavioral self-regulation in European samples [57, 58]. Our own interrater reliability test based on video scoring of 18 children prior to data collection showed an intraclass correlation coefficient (ICC) of 1.00 for the HTKS. The iPad-based Early Years Toolbox (EYT) was used to assess three fundamental constructs of EF, i.e., inhibition, working memory, and cognitive flexibility [6, 11, 12, 17], in addition to early expressive vocabulary and early mathematical skills, referred to hereafter as “vocabulary” and “numeracy”. The EYT has shown strong reliability, convergent validity with existing measures, and developmental sensitivity [59, 60].

All measures of cognitive function and academic skills were performed at the preschools, individually in a room without disturbances, instructed by a trained assessor. The testing was divided into two blocks of three tests (in standardized order), with a 15-min (minimum) break after the first block. Block 1: Inhibition, numeracy, and self-regulation; and Block 2: Working memory, cognitive flexibility, and vocabulary. All tasks were instructed in Norwegian, and the total test duration varied from 30–45 min, depending on the children’s performance.

We measured *behavioral self-regulation* using the HTKS, which assesses a child’s ability to integrate and apply EF to control and direct actions despite instructions that generate contrary impulses [57]. The children were asked to remember instructions, inhibit the automatic responses generated, and instead execute gross motor movements in line with the task goals (the opposite of the dominant response). The task consisted of three blocks of 10 trials each, e.g., the children were instructed to touch their head when asked to “touch your toes”. The complexity of the rules increased with each new block. Children scored 2 points for executing the correct response (opposite of the instruction), 1 point for self-correction to the correct response (after an initial incorrect response), and 0 points for incorrect response. ≥ 4 points on a block was the criterion for advancing to the next block. Possible scores ranged from 0 to 60, where a higher score indicated better self-regulation.

We measured *response inhibition* using the Go/No-Go task, asking the children to catch the fish (Go-trial, 80%) by tapping the screen when they see a fish, and to avoid the shark (No-Go trial, 20%) by not tapping the screen when they see a shark. The task started with instructions and a practice round, followed by 75 stimuli divided evenly into three 1-min test blocks separated by a short break with repeated instructions. The animated stimulus (fish or shark) was visible for 1.5 s and separated by a 1-s interstimulus interval. Responses < 0.3 s were unlikely to be initiated in response to the stimulus and were therefore considered anticipations and removed. The outcome was an impulse control score, representing the average score on all Go-trials multiplied by the average score on all No-Go-trials. Possible scores ranged from 0.00 to 1.00, where a higher score indicated better inhibition.

We measured *visual-spatial working memory* using the Mr. Ant task, asking the children to remember the spatial locations of an increasing number of “sticker(s)” located on Mr. Ant’s body. The task started with instructions and a practice round, followed by three trials (or two trials if correct on the first two) at level one (remembering one sticker), with each subsequent level increasing in difficulty by adding one sticker. The task lasted until completing level eight or failure on all three trials at the current level. Each passed level was awarded 1 point, plus 1/3 of a point for all correct trials thereafter. Possible scores ranged from 0 to 8, where a higher score indicated better working memory.

We measured* cognitive flexibility* using the Card Sorting task, asking the children to sort cards of red and blue rabbits and boats by either color or shape, into two locations (identified by a blue rabbit and a red boat). The children must switch between rules as the card sorting rules alternate. The task started with instructions and a practice round, followed by three levels of six trials each. On level one, children were asked to sort cards by color, and on level two by shape. If children performed ≥ 5 out of 6 correct trials on both levels, they moved on to level three and were then asked to sort cards by color if they were surrounded by a black border or by shape if there was no black border. Each correct sort on level two (when the switch trials began) and three were awarded 1 point. If the accuracy on level two was higher than that on level one, the values from level one replaced the values from level two. Possible scores ranged from 0 to 12, where a higher score indicated better cognitive flexibility.

We measured *expressive vocabulary* using the Expressive Vocabulary task, which consists of 54 items with increasing difficulty, asking the children to verbally produce a correct label for the depicted nouns and verbs. The task continued until the child completed all items or automatically stopped because they answered six items in a row incorrectly. Each correct item was awarded 1 point (we only accepted words pronounced in Norwegian). Possible scores ranged from 0 to 54, where a higher score indicated better vocabulary.

We measured* numeracy* using the Early Numeracy task, which consists of 79 items of increasing difficulty that pertain to numerical concepts, spatial and measurement concepts, counting subsets, matching digits and quantities, number, ordinality, cardinality, subitizing, patterning, numerical word problems, and equations. The starting point was item 1, 11, and 21 for children aged 3, 4, and 5 years, respectively. If the child provided three consecutive incorrect responses to begin with (for those who started on item 11 or 21), the app returned to the prior starting point. After five consecutive incorrect responses, the task was automatically stopped. Each correct item across the entire task was awarded 1 point. Possible scores ranged from 0 to 79, where a higher score indicated better numeracy.

### Potential covariates

Sex, age, and parental education level were reported by parent(s) or guardian(s) via questionnaire. Parental education level was the highest education level of the mother or father, categorized as ≤ upper secondary school, < 4 years at university/college, and ≥ 4 years at university/college. Body mass (weight; 0.1 kg) was measured using an electronic scale (Seca 899, SECA GmbH, Hamburg, Germany) with children wearing light clothing. Stature (height; 0.1 cm) was measured using a transportable Seca 217 (SECA GmbH, Hamburg, Germany). BMI (kg·m^−2^) was calculated. Children were categorized as normal weight (including underweight), overweight, or obese according to the age- and sex-specific BMI cut-points established by Cole et al. [61] for descriptive purposes.

### Statistical analyses

The children’s characteristics are presented as means, proportions, and standard deviations (SDs). We tested for differences in characteristics between included and excluded children using linear regression, in which models for PA and SED were adjusted for wear time.

In the primary analysis, longitudinal associations between movement behaviors and change in cognitive function and academic skills were analyzed using multivariate pattern analysis, that is, partial least squares (PLS) regression, equivalent to its previous application to accelerometer data [38]. This approach can handle completely multicollinear variables by decomposing the explanatory variables into orthogonal linear combinations (PLS components) while simultaneously maximizing the covariance with the outcome variable [62]. We created models including the entire triaxial PA intensity spectrum, screen time, and sleep at baseline as explanatory variables, and change in self-regulation, inhibition, working memory, cognitive flexibility, vocabulary, or numeracy as outcome variables. In a sensitivity analysis, we replaced parent-reported sleep with accelerometry-derived sleep. In all models, the explanatory and outcome variables were adjusted for sex, age, BMI, and parental education level using covariate projection [63]. The PA data were additionally adjusted for wear time, and the outcome variables were additionally adjusted for group (intervention/control) and baseline levels. Models were cross-validated using Monte Carlo resampling [64] with 1,000 repetitions by repeatedly and randomly keeping 50% of the subjects as an external validation set. The model with the number of PLS components leading to the lowest prediction error was retained, followed by target projection [65, 66]. If explanatory variables were significantly associated with outcomes, we reported explained variance (R^2^) of the model and the strength of the associations for each explanatory variable, using multivariate correlation coefficients with 95% confidence intervals (CIs) [37]. Multivariate pattern analysis, including adjustment using covariate projection, was performed using R Statistical Software (v4.3.1; R Core Team 2023) and the *mvpa* (v0.2.2) package [67].

In secondary analyses (with adjustment for potential covariates as detailed above), we tested interactions for sex with all explanatory variables for each outcome using full factorial models in linear regression. Association patterns were explored for boys and girls using multivariate pattern analysis if interactions were indicated. For comparison with previous studies on movement behaviors, we also performed linear regression analyses for separate explanatory variables (PA using cut-points defined by Evenson et al. [47], screen time, and sleep) with outcomes. Furthermore, we considered the combined influence of compliance with all three recommendations of the movement behavior guidelines (PA, screen time, and sleep) at baseline (yes, no) with change in outcomes at follow-up. Interaction analyses and linear regression analyses were performed using the SPSS software (v.28.0; IBM SPSS Statistics for Windows, Armonk, NY: IBM Corp., United States). Associations were considered statistically significant if* p* ≤ 0.05.

## Results

### Children’s characteristics

**Fig. 1:**
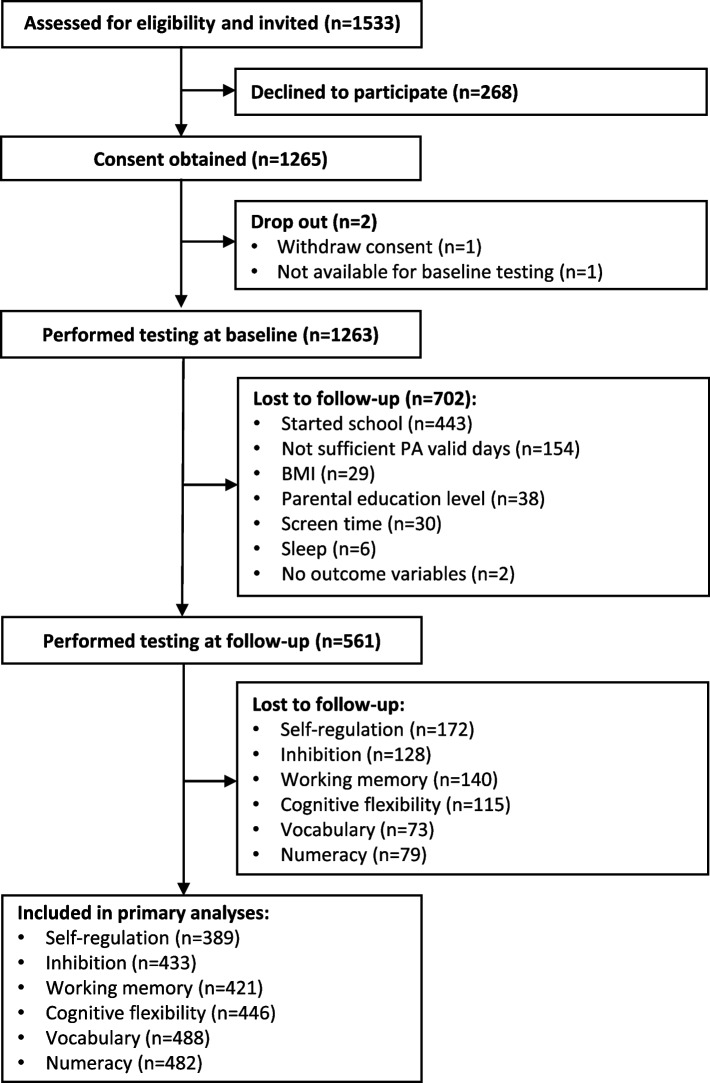
Flow of recruitment of the children and missing data (*n* = children)

Of the 820 eligible children aged 3–4 years, 561 (53% boys) provided valid data on all movement behaviors, covariates, and at least one of the outcome variables (*n* = 389–488 across outcomes, Fig. 1). Children’s movement behavior characteristics and covariates are shown in Table 1, and children’s scores on cognitive and academic outcomes at baseline and follow-up are shown in Table 2. The main reasons for missing data were that children had started school, parents did not complete the sleep questionnaire, children had insufficient accelerometry wear time or did not want to participate in one or more cognitive or academic tasks/tests, or task(s) were unsuitable for children due to their young age, language barriers, and/or other mental/physical disabilities. The included 3–4-year-old children did not differ from the excluded children regarding SED (*p* = 0.241), PA (*p* = 0.124–0.462), screen time (*p* = 0.736), self-regulation (*p* = 0.779), working memory (*p* = 0.061), or cognitive flexibility (*p* = 0.156), but had less sleep (*p* = 0.018) and higher scores on inhibition (*p* = 0.009), vocabulary (*p* = 0.003), and numeracy (*p* = 0.002).

**Table 1:** Children’s characteristics for movement behaviors and covariates at baseline. Values are means (SDs) or proportions

	n	Baseline
Age (years)	561	3.8 (0.6)
BMI (kg*m^−2^)	561	16.4 (1.4)
Weight status (%)^a^		
Normal weight	472	84.1
Overweight	76	13.5
Obese	13	2.3
Parental education level (%)		
≤ Upper secondary school	134	23.9
< 4 years University/college	161	28.7
≥ 4 years University/college	266	47.4
Physical activity and sedentary behavior^b^		
Wear time (min/day)	561	761 (67)
Total PA (cpm)	561	659 (143)
SED (min/day)	561	536 (62)
LPA (min/day)	561	153 (21)
MPA (min/day)	561	37 (7)
VPA (min/day)	561	35 (10)
MVPA (min/day)	561	72 (16)
Meeting PA guidelines (%)	439	78.3
Screen time		
Screen time (min/day)	561	91.8 (56.9)
Meeting screen time guidelines (%)	242	43.1
Sleep^c^		
Sleep (hours/day)	561	11.1 (0.7)
Meeting sleep guidelines (%)	549	97.9

*BMI* Body mass index, *SED* Sedentary behavior, *LPA* Light-intensity physical activity, *MPA* Moderate-intensity physical activity, *VPA* Vigorous-intensity physical activity, *MVPA* Moderate-to- vigorous-intensity physical activity, *cpm* counts per minute

^a^Defined by the Cole et al. (2000) criteria

^b^Defined by the Evenson et al. (2008) cut-points applied to the vertical axis

^c^Parent-reported sleep. Meeting PA guidelines: ≥ 180 min/day of total PA and ≥ 60 min/day of MVPA, meeting screen time guidelines: ≤ 1 h/day of screen time, and meeting sleep guidelines: ≥ 10 h/day of sleep

**Table 2:** Children’s scores for cognitive and academic outcomes at baseline and follow-up

	n	Baseline	Follow-up
Self-regulation	389	6.9 (12.4)	30.7 (18.5)
Inhibition	433	0.47 (0.24)	0.77 (0.18)
Working memory	421	1.1 (0.9)	2.0 (0.9)
Cognitive flexibility	446	3.5 (4.0)	7.1 (3.7)
Vocabulary	488	20.3 (9.0)	32.9 (8.6)
Numeracy	482	21.0 (11.8)	43.5 (13.9)

Values are means (SDs). Possible score ranges: Self-regulation, 0–60; Inhibition, 0.00–1.00; Working memory, 0–8; Cognitive flexibility, 0–12; Vocabulary, 0–54; Numeracy, 0–74

### Multivariate pattern analysis

**Fig. 2:**
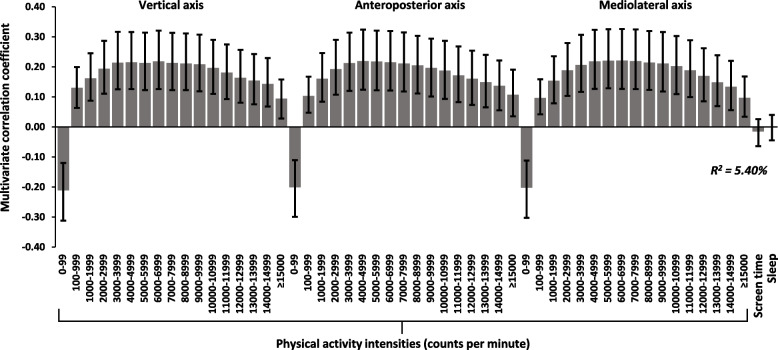
The longitudinal multivariate 24-h movement behavior pattern associated with inhibition in girls. Movement behaviors include triaxial PA intensity data (counts per minute), screen time (min/day), and sleep (hours/day). Results are reported as multivariate correlation coefficients and 95% CIs. In the model (PLS regression), all variables are adjusted for age, BMI, and parental education level. PA and SED are additionally adjusted for wear time, and inhibition for group (intervention/control) and baseline levels

**Fig. 3:**
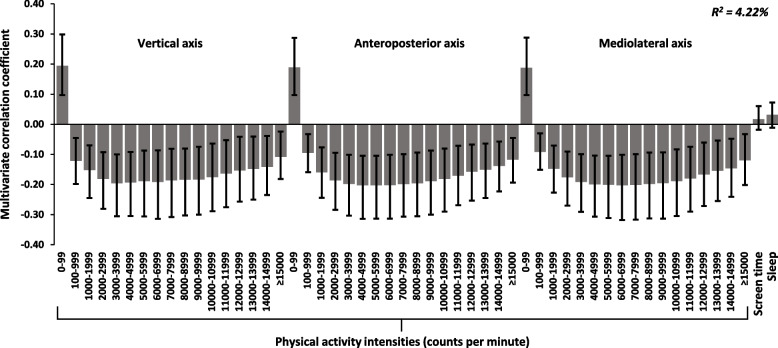
The longitudinal multivariate 24-h movement behavior pattern associated with self-regulation in girls. Movement behaviors include triaxial PA intensity data (counts per minute), screen time (min/day), and sleep (hours/day). Results are reported as multivariate correlation coefficients and 95% CIs. In the model (PLS regression), all variables are adjusted for age, BMI, and parental education level. PA and SED are additionally adjusted for wear time, and self-regulation for group (intervention/control) and baseline levels

Interrelations between change in outcomes variables were weak (standardized associations ≤ 0.28) in the total group (Additional Table 1) and for boys and girls separately (Additional Table 2). In primary analyses, we found no significant associations for the PA intensity spectrum, screen time, or sleep at baseline with change in cognitive or academic outcomes at follow-up. Sensitivity analyses showed no significant associations when replacing parent-reported sleep with accelerometry-derived sleep. In subgroup analyses, we found significant interactions with sex for the PA intensity spectrum with change in inhibition (37 out of 51 interactions across three axes) and self-regulation (6 out of 51 interactions across three axes) but not for screen time or sleep (Additional Table 3). When boys and girls were analyzed separately, significant association patterns were found for change in inhibition (R^2^ = 5.40%) and self-regulation (R^2^ = 4.22%) in girls. For inhibition, associations were negative for 0–99 cpm and positive for all intensities ≥ 100 cpm (Fig. 2). For self-regulation, associations were positive for 0–99 cpm and negative for all intensities ≥ 100 cpm (Fig. 3). Associations for screen time and sleep were non-significant in both analyses.

### Linear regression analysis

We found no significant associations with change in cognitive or academic outcomes at follow-up for any of the explanatory variables analyzed separately (Additional Table 4) or for complying with all movement behavior guidelines at baseline (32% of the children) (Additional Table 5).

## Discussion

In this study, we examined longitudinal associations for 24-h movement behaviors using the entire PA intensity spectrum, screen time, and sleep with preschool children’s development of self-regulation, EF, vocabulary, and numeracy. We found no significant associations in primary analyses. However, secondary analyses showed that less SED and more PA were favorably associated with inhibition, and that more SED and less PA were favorably associated with self-regulation in girls.

To the best of our knowledge, no previous studies have examined longitudinal associations for all movement behaviors combined using continuous variables, with preschoolers’ development of cognitive function and/or academic skills. As such, we could only partially compare our findings with previous studies. Our general lack of significant longitudinal associations for PA is in accordance with the findings of Verswijveren et al. [28], who found no associations for SED, LPA, or MVPA with inhibition, working memory, or general cognitive ability score over 12 months in preschoolers. Similarly, Zhang et al. [29] found no associations for participation in organized or non-organized PA with inhibition or working memory after 6 and 12 months. In contrast, McNeill et al. [32, 68] reported significant positive associations for VPA and for compliance with PA guidelines with shifting performance (i.e. cognitive flexibility) after 12 months, which were not replicated in the present study. Also somewhat in contrast, Zhang et al. [29] found positive associations for participation in PA with intellectual ability (i.e., language development, inductive reasoning, and perceptual speed); however, the lack of differentiation among PA intensities does not allow for thorough comparison.

Due to high proportions of guideline compliances, we did not analyze associations for complying with each movement behavior guideline separately. The contrasting findings by Zhang et al. [29] and McNeill et al. [68] may be explained by their use of parent-reported PA and not including the entire PA spectrum, respectively. Although parent-reported PA may have poor validity and reliability [69], accelerometry-derived PA may be influenced by seasonal variation [44] and may capture both meaningful (e.g., structured PA, active play, exploration) and incidental PA (e.g., simple actions) [27, 70]. Compared with incidental PA, meaningful PA is more likely to challenge cognitive skills and subsequently be more beneficial for cognitive development [71, 72]; hence, the reliability of device-based PA also needs careful consideration.

Our findings suggest that associations for the PA intensity spectrum with inhibition and self-regulation may be moderated by sex. For inhibition, findings were consistent across intensities and axes, showing significant interactions for 37 of the 51 PA variables, whereas findings were less consistent and convincing for self-regulation, showing significant interactions for 6 of the 51 PA intensity variables. While no associations for these outcomes were found for boys, we found positive associations for PA (negative for SED) with inhibition and negative associations for PA (positive for SED) with self-regulation among girls. The positive association for PA with inhibition contributes to a field of still unclear and inconsistent evidence across the entire developmental age [73] and specifically response inhibition at preschool age [74]. The negative association for PA with self-regulation is challenging to explain, when considering that response inhibition seems to be associated with and underly self-regulation [11, 75]. However, these variables were not associated in our study, neither in the total sample nor in girls or boys separately.

The divergent associations for PA/SED with inhibition and self-regulation, may have several possible explanations. First, in early childhood, inconsistencies may be due to different motor demands of the cognitive tasks [76], with the HTKS self-regulation task involving a more complex gross-motor response in contrast to an easier fine-motor response in the Go/No-Go inhibition task. However, neither tasks are considered notably motor demanding, thus, it is difficult to explain the different associations by transferability to the cognitive tasks. Second, substantial increases over the 18-month period were found for both HTKS and the inhibition task, which means that associations with movement behaviors could be influenced by large developmental and/or learning effects. Yet, it is not clear to us how this potentially could induce differential effects for associations with the two outcomes. Third, the HTKS task is validated in children from the age of 4 years, meaning that there could be a floor effect for younger children. A floor effect for HTKS is commonly seen in the literature and a revised version including easier tasks has recently been developed to address this issue [77, 78]. However, we do not see how a potential floor effect could confound associations with movement behaviors. Moreover, the distribution of scores were similar for boys and girls and change scores were normally distributed in both subgroups, which argue against such an explanation for the differential association pattern found in boys and girls. Fourth, since the divergent associations emerged only in girls, they might reflect gender preferences in types of PA and play characteristics. Girls are more sedentary and less physically active than boys [79, 80], and tend to prefer more social, verbal, and less intensive play [81, 82]. Thus, girls being more physically active may spent less time on sedentary activities that are beneficial for self-regulation. For example, solving puzzles or reading books indoors may demand interference control (i.e., selective attention and inhibition of thoughts and emotions) beyond inhibition at the level of behavior, which may be better reflected in the self-regulation task compared to the response inhibition task [11]. Moreover, sedentary behaviors may provide opportunities for reducing stress, increase concentration, and establish peer relations, which may also be beneficial for especially self-regulation [83]. However, since the present study has no information on qualitative PA or SED characteristics, these hypotheses are only speculative and comparison with previous studies is difficult since they lack subgroup analyses.

In contrast to our null findings, excessive screen time has been negatively associated with development of cognitive and academic skills in children and adolescents [19, 22, 30]. In preschoolers, Zhang et al. [29] showed no associations for continuous screen time variables with inhibition, working memory, or intellectual ability after 6 and 12 months, which is in accordance with the findings of the present study. However, Zhang et al. [29] also found that meeting the screen time guidelines was associated with better intellectual ability. These different findings for continuous and categorical variables may indicate a certain threshold of screen time that, when exceeded, may negatively impact cognitive development. Moreover, while we did not assess content and context of screen time, several systematic reviews have showed beneficial associations for certain aspects/types of screen time with cognitive and/or academic-related outcomes [19, 30]. For example, Madigan et al. [30] found that high-quality screen time (i.e., educational programs, slow-paced, and co-viewed) was positively associated with language development and Liu et al. [19] found benefits of digital educational games on early literacy skill and creative thinking abilities.

We found no association for sleep with cognitive or academic outcomes, which contrasts with favorable longitudinal associations shown with both cognitive and academic outcomes during preschool years [84, 85]. Meeting the sleep guidelines at preschool age has also been associated with better reading, spelling, writing, language, and numeracy four years later [31]. As previously discussed, the present study did not perform analyses for compliance with guidelines separately; however, the contrasting findings may be explained by other methodological considerations. Previous studies using continuous variables had small sample sizes, Bernier et al. [85] examined sleep in infancy with EF at preschool age, and Hinkley et al. [31] examined sleep in preschoolers with academic outcomes at school age. Furthermore, sleep diary data in Bernier et al. [85] and sleep questionnaires in Jung et al. (2009) and Hinkley et al. (2020) typically report time in bed as opposed to time asleep [49, 86]. Hence, the present study also examined associations using accelerometry-derived sleep, suggested to be a viable alternative for capturing both nighttime sleep patterns and naps over several days [50, 51]. We used an algorithm for accelerometry-derived sleep developed for young children [55], however, the alternative sleep assessment did not change our results. Since this algorithm has not been validated against polysomnography, which is accepted as the gold standard for assessing sleep [87, 88], we cannot yet establish which assessment most accurately captures young children’s sleep.

In accordance with previous studies [31, 32], we found no longitudinal associations for meeting all 24-h movement behavior guidelines with development of preschool children’s cognitive or academic skills. In other words, using continuous or dichotomized variables may seem to provide similar results regarding the combined influence of movement behaviors for change in cognitive function and academic skills. Generally, we emphasize the large number of associations examined in previous studies, where only a few associations are significant. For example, in the study by McNeill et al. [32], only 5 of their 126 examined associations for compliance with movement behaviors with EF and psychosocial health (cross-sectionally and longitudinally) were statistically significant. Similarly, in the present study, only 2 of 72 associations (primary and secondary analyses) were statistically significant, underlining an important consideration with regards to trustworthiness and impact of findings.

### Strengths and limitations

The main strengths of this study were the inclusion of the entire PA spectrum, screen time, and sleep for examining longitudinal association patterns for all 24-h movement behaviors with change in cognitive function and academic skills using multivariate pattern analysis. Furthermore, we included a relatively large sample of preschoolers, and analyzed both the separate and combined influences of movement behaviors.

A limitation was the large drop-out from baseline to follow-up, however, this was mainly due to children reaching the age for schooling, which does not imply a risk of selection bias. Nonetheless, the included children differed from the excluded children regarding sleep, inhibition, vocabulary, and numeracy, thus, our analytic sample may be less representative than the entire sample for these variables. Our sensitivity analysis for accelerometry-derived sleep had reduced sample size and statistical power, however, this approach did not impact our results. We consider the use of accelerometers a strength of the study, however, accelerometers are not able to correctly capture certain activities (e.g., crawling, climbing, cycling, and rolling), and cannot provide qualitative information about the content and context of PA and SED. The same limitation applies to our screen time assessment, which did not separate educational from noneducational screen use, which could be relevant for associations with cognitive and academic outcomes. Findings from the present study were drawn from an intervention study on preschool staff professional development in PA [41] and could potentially be biased by intervention effects. We found few and weak effects of the intervention on cognitive and academic outcomes [89], however, to account for this, all outcome variables were adjusted for group (intervention/control). Finally, a longer follow-up period and larger sample size would have strengthened our study.

## Conclusions

The present study found no longitudinal associations for 24-h movement behaviors with change in cognitive function or academic skills in preschool-aged children. However, in girls, less SED and more PA was associated with better inhibition, and more SED and less PA was associated with better self-regulation. We recommend that future studies follow children for a longer period and further examine the role of sedentary behaviors, including screen time, and their possible contributions to different domains of young children’s cognitive function and academic skills.

## Supplementary Information

Additional file 1. Additional Table 1. Standardized associations between outcome variables in the total sample. Additional Table 2. Standardized associations between outcome variables in boys (above principal diagonal) and girls (below principal diagonal). Additional Table 3. P-values for interactions with sex for the PA intensity spectrum, screen time, and sleep at baseline with change in cognitive function and academic skills at follow-up. Additional Table 4. Standardized associations (95% confidence intervals) for movement behaviors at baseline with change in cognitive function and academic skills at follow-up. Additional Table 5. Standardized associations (95% confidence intervals) for compliance with PA, screen time, and sleep guidelines at baseline with change in cognitive function and academic skills at follow-up

## Data Availability

The dataset used in the current study is available from the corresponding author upon reasonable request.

## Abbreviations

ACTNOW: Active Learning Norwegian Preschool(er)s
BMI: Body mass index
CI: Confidence interval
cpm: Counts per minute
EF: Executive function
EYT: Early Years Toolbox
HTKS: Head-Toes-Knees-Shoulders
ICC: Intraclass correlation coefficient
LPA: Light-intensity physical activity
MPA: Moderate-intensity physical activity
MVPA: Moderate- to vigorous-intensity physical activity
PA: Physical activity
PLS: Partial least squares
SD: Standard deviation
SED: Sedentary behavior
TPA: Total physical activity
VPA: Vigorous physical activity
WHO: World Health Organization
WMA: World Medical Association's Declaration
