# Supplementary material for: Longitudinal associations between preschoolers’ 24-h movement behaviors and development of self-regulation, executive function, vocabulary, and numeracy
Source: BMC Public Health. 2025 Oct 6;25:3241. doi: 10.1186/s12889-025-24605-z (PMC12498441; doi:10.1186/s12889-025-24605-z)
Supplement: Supplementary file 1 — Additional file 1. Additional Table 1. Standardized associations between outcome variables in the total sample. Additional Table 2. Standardized associations between outcome variables in boys (above principal diagonal) and girls (below principal diagonal). Additional Table 3. P-values for interactions with sex for the PA intensity spectrum, screen time, and sleep at baseline with change in cognitive function and academic skills at follow-up. Additional Table 4. Standardized associations (95% confidence intervals) for movement behaviors at baseline with change in cognitive function and academic skills at follow-up. Additional Table 5. Standardized associations (95% confidence intervals) for compliance with PA, screen time, and sleep guidelines at baseline with change in cognitive function and academic skills at follow-up [file 12889_2025_24605_MOESM1_ESM.pdf]

Additional Table 1. Standardized associations between outcome variables in the total sample.

	1	2	3	4	5	6
1. Self-regulation	-					
2. Inhibition	0.05	-				
3. Working memory	**0.12**	**0.16**	-			
4. Cognitive flexibility	**0.27**	**0.18**	0.04	-		
5. Vocabulary	**0.19**	**0.11**	0.00	0.06	-	
6. Numeracy	**0.19**	**0.13**	**0.12**	0.08	**0.14**	-

All variables were adjusted for sex, age, BMI, parental education level, group, and baseline levels; Significant associations at p ≤ 0.05 are highlighted in boldface.

Additional Table 2. Standardized associations between outcome variables in boys (above principal diagonal) and girls (below principal diagonal).

	1	2	3	4	5	6
1. Self-regulation	-	0.02	0.13	**0.28**	**0.24**	**0.22**
2. Inhibition	0.01	-	**0.15**	0.13	0.13	**0.18**
3. Working memory	0.01	**0.16**	-	0.02	-0.01	**0.20**
4. Cognitive flexibility	**0.27**	**0.27**	0.08	-	**0.16**	0.12
5. Vocabulary	0.14	0.09	0.02	-0.07	-	**0.13**
6. Numeracy	**0.16**	0.06	0.02	0.04	**0.15**	-

All variables were adjusted for age, BMI, parental education level, group, and baseline levels; Significant associations at p ≤ 0.05 are highlighted in boldface.

Additional Table 3. P-values for interactions with sex for the PA intensity spectrum, screen time, and sleep at baseline with change in cognitive function and academic skills at follow-up.

	**Self-regulation**	**Inhibition**	**Working memory**	**Cognitive flexibility**	**Vocabulary**	**Numeracy**
	p	*p*	*p*	*p*	*p*	*p*
**X-Axis**						
0–99 cpm	0.063	**0.002**	0.992	0.504	0.074	0.165
100–999 cpm	0.221	0.123	0.716	0.227	0.229	0.102
1000–1999 cpm	0.088	**0.010**	0.617	0.414	0.064	0.291
2000–2999 cpm	**0.040**	**0.001**	0.565	0.485	0.095	0.511
3000–3999 cpm	**0.035**	**<0.001**	0.955	0.673	0.160	0.479
4000–4999 cpm	0.076	**0.001**	0.994	0.969	0.142	0.311
5000–5999 cpm	0.220	**0.003**	0.834	0.806	0.078	0.249
6000–6999 cpm	0.334	**<0.001**	0.606	0.572	**0.041**	0.249
7000–7999 cpm	0.449	**0.002**	0.761	0.701	**0.030**	0.269
8000–8999 cpm	0.463	**0.002**	0.836	0.525	**0.024**	0.306
9000–9999 cpm	0.348	**0.004**	0.916	0.499	**0.016**	0.251
10000–10999 cpm	0.539	**0.021**	0.810	0.368	**0.036**	0.176
11000–11999 cpm	0.331	**0.047**	0.493	0.575	**0.023**	0.178
12000–12999 cpm	0.431	0.125	0.485	0.521	**0.048**	0.200
13000–13999 cpm	0.428	0.361	0.400	0.741	0.057	0.379
14000–14999 cpm	0.404	0.738	0.361	0.982	0.177	0.692
≥ 15000 cpm	0.071	0.915	0.498	0.563	0.851	0.082
**Y-Axis**						
0–99 cpm	0.144	**0.004**	0.703	0.896	0.485	0.362
100–999 cpm	0.413	0.315	0.797	0.510	0.648	0.833
1000–1999 cpm	**0.031**	0.086	0.880	0.307	0.389	0.449
2000–2999 cpm	**0.035**	**0.002**	0.668	0.643	0.411	0.291
3000–3999 cpm	0.058	**<0.001**	0.502	0.917	0.493	0.433
4000–4999 cpm	0.083	**<0.001**	0.514	0.585	0.576	0.648
5000–5999 cpm	0.094	**<0.001**	0.492	0.567	0.545	0.780
6000–6999 cpm	0.128	**<0.001**	0.748	0.666	0.582	0.702
7000–7999 cpm	0.133	**<0.001**	0.963	0.725	0.475	0.725
8000–8999 cpm	0.081	**0.001**	0.798	0.712	0.549	0.928
9000–9999 cpm	0.077	**<0.001**	0.531	0.909	0.626	0.672
10000–10999 cpm	**0.035**	**0.006**	0.609	0.827	0.626	0.722
11000–11999 cpm	0.143	**0.007**	0.554	0.792	0.770	0.852
12000–12999 cpm	0.104	**0.009**	0.741	1.000	0.851	0.903
13000–13999 cpm	0.070	**0.036**	0.998	0.986	0.980	0.763
14000–14999 cpm	0.068	0.070	0.387	0.722	0.924	0.969
≥ 15000 cpm	0.127	0.290	0.586	0.591	0.949	0.787
**Z-Axis**						
0–99 cpm	0.186	**0.003**	0.728	0.969	0.484	0.510
100–999 cpm	0.459	0.275	0.864	0.846	0.752	0.514
1000–1999 cpm	0.083	0.053	0.986	0.851	0.397	0.294
2000–2999 cpm	**0.040**	**0.001**	0.565	0.485	0.095	0.511
3000–3999 cpm	0.053	**0.001**	0.559	0.914	0.937	0.950
4000–4999 cpm	0.129	**<0.001**	0.481	0.771	0.834	0.941
5000–5999 cpm	0.152	**<0.001**	0.472	0.644	0.841	0.989
6000–6999 cpm	0.237	**<0.001**	0.431	0.377	0.805	0.867
7000–7999 cpm	0.191	**<0.001**	0.567	0.410	0.986	0.769
8000–8999 cpm	0.219	**<0.001**	0.511	0.562	0.885	0.919
9000–9999 cpm	0.107	**0.003**	0.757	0.680	0.888	0.998
10000–10999 cpm	0.172	**0.007**	0.999	0.695	0.880	0.732
11000–11999 cpm	0.150	**0.016**	0.833	0.787	0.974	0.645
12000–12999 cpm	0.073	**0.042**	0.569	0.947	0.868	0.657
13000–13999 cpm	0.051	0.076	0.525	0.712	0.641	0.561
14000–14999 cpm	0.113	0.170	0.539	0.633	0.675	0.305
≥ 15000 cpm	0.064	0.218	0.423	0.570	0.664	0.053
**Screen time**	0.183	0.906	0.366	0.795	0.270	0.336
**Sleep**	0.489	0.411	0.891	0.391	0.789	0.219

cpm = counts per minute. Significant associations at $p \leq 0.05$ are highlighted in boldface.

Additional Table 4. Standardized associations (95% confidence intervals) for movement behaviors at baseline with change in cognitive function and academic skills at follow-up.

	Outcome at follow-up					
	**Self-regulation** n=389	**Inhibition** n=433	**Working memory** n=421	**Cognitive flexibility** n=446	**Vocabulary** n=488	**Numeracy** n=482
Total PA (cpm)	-0.04 (-0.12–0.05)	0.04 (-0.06–0.13)	0.03 (-0.07–0.12)	0.03 (-0.07–0.12)	0.08 (-0.01–0.10)	0.01 (-0.05–0.06)
SED (min/day)	0.07 (-0.06–0.28)	0.01 (-0.17–0.20)	-0.04 (-0.28–0.10)	-0.03 (-0.24–0.13)	-0.06 (-0.18–0.04)	0.06 (-0.04–0.19)
LPA (min/day)	-0.04 (-0.13–0.06)	-0.03 (-0.13–0.08)	0.04 (-0.06–0.15)	0.03 (-0.07–0.13)	0.05 (-0.03–0.09)	-0.08 (-0.13–0.01)
MPA (min/day)	-0.07 (-0.15–0.02)	-0.02 (-0.12–0.08)	0.05 (-0.05–0.15)	0.03 (-0.07–0.13)	0.05 (-0.02–0.09)	-0.03 (-0.09–0.04)
VPA (min/day)	-0.08 (-0.15–0.02)	0.04 (-0.06–0.13)	0.03 (-0.07–0.12)	0.01 (-0.08–0.10)	0.05 (-0.02–0.09)	-0.00 (-0.06–0.06)
MVPA (min/day)	-0.08 (-0.15–0.02)	0.01 (-0.08–0.11)	0.04 (-0.06–0.14)	0.02 (-0.08–0.12)	0.05 (-0.02–0.09)	-0.01 (-0.07–0.05)
Screen time (min/day)	0.02 (-0.06–0.10)	-0.05 (-0.14–0.04)	0.05 (-0.04–0.14)	-0.03 (-0.12–0.06)	-0.07 (-0.09–0.01)	-0.01 (-0.06–0.05)
Sleep (min/day)	0.01 (-0.07–0.09)	0.02 (-0.07–0.11)	0.02 (-0.07–0.11)	0.02 (-0.07–0.11)	0.01 (-0.05–0.06)	0.01 (-0.05–0.06)

cpm = counts per minute; SED = sedentary time; LPA = light-intensity physical activity; MPA = moderate-intensity physical activity; VPA = vigorous-intensity physical activity; MVPA = moderate- to vigorous-intensity physical activity, according to cut points defined by the Evenson et al. (Evenson et al., 2008) applied to the vertical axis. All variables are adjusted for sex, age, BMI, and parental education level. PA and SED are additionally adjusted for wear time, and outcomes are additionally adjusted for group and baseline levels.

Additional Table 5. Standardized associations (95% confidence intervals) for compliance with PA, screen time, and sleep guidelines at baseline with change in cognitive function and academic skills at follow-up.

	**n**	**PA, Screen time, and Sleep**
Self-regulation	389	-0.07 (-0.28–0.06)
Inhibition	433	0.05 (-0.08–0.29)
Working memory	421	-0.04 (-0.27–0.11)
Cognitive flexibility	446	0.03 (-0.12–0.25)
Vocabulary	488	0.04 (-0.06–0.16)
Numeracy	482	-0.04 (-0.17–0.06)

PA = physical activity (cut points defined by Evenson et al. (2008)). Outcomes are adjusted for sex, age, BMI, parental education level, group, and baseline levels.
